# Supplementary figures and images for: Enhancer Clusters Drive Type I Interferon-Induced TRAIL Overexpression in Cancer, and Its Intracellular Protein Accumulation Fails to Induce Apoptosis
Source: Cancers (Basel). 2023 Feb 3;15(3):967. doi: 10.3390/cancers15030967 (PMC9913803; doi:10.3390/cancers15030967)

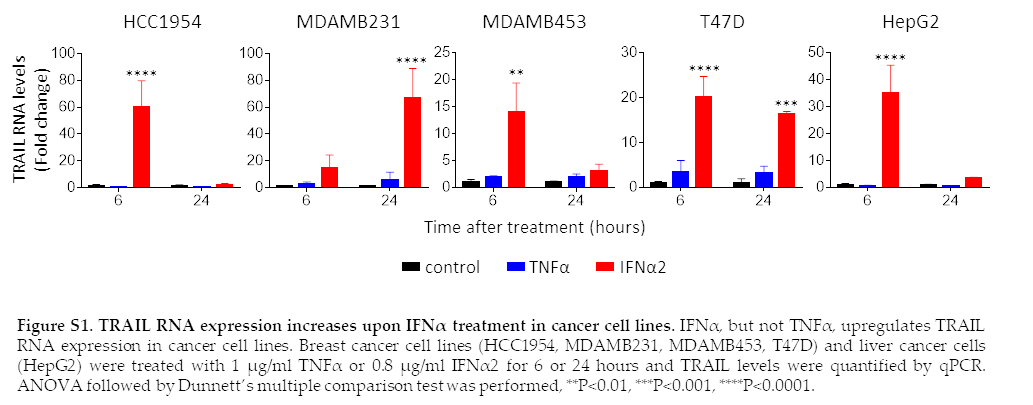

Supplement: Supplementary file 1 [file cancers-15-00967-s001.zip › Figure S1.tif]

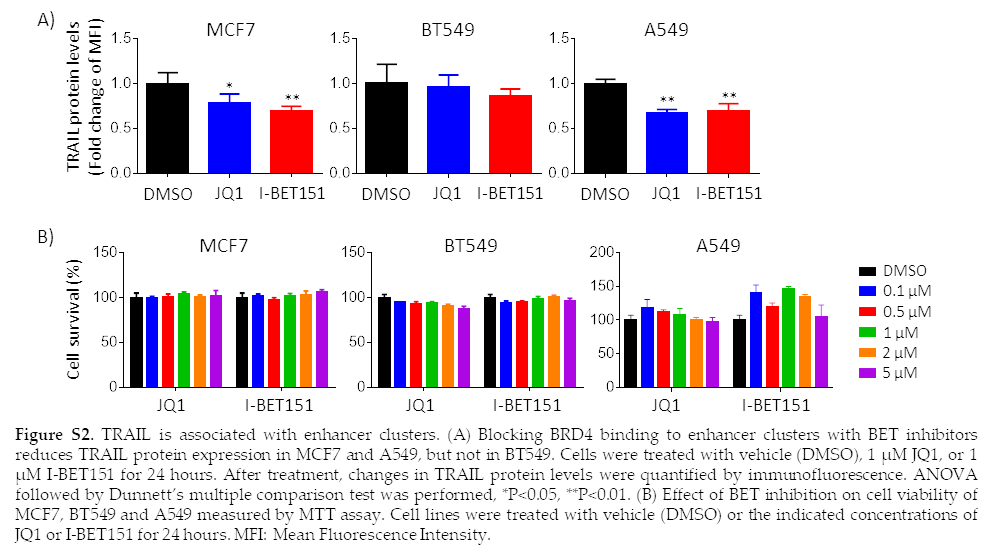

Supplement: Supplementary file 1 [file cancers-15-00967-s001.zip › Figure S2.tif]

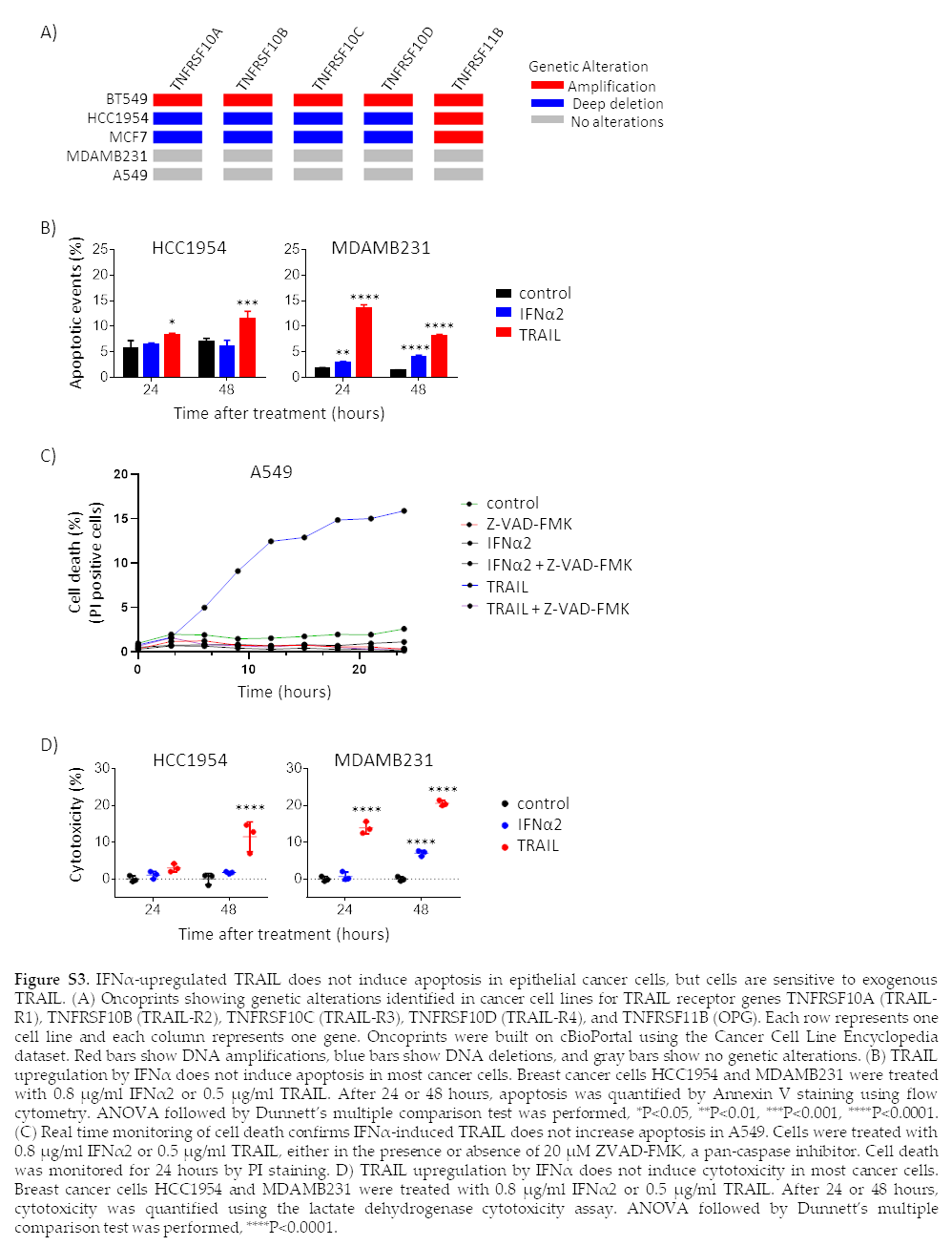

Supplement: Supplementary file 1 [file cancers-15-00967-s001.zip › Figure S3.tif]
